# A Study on Consumers’ Visual Image Evaluation of Wrist Wearables

**DOI:** 10.3390/e23091118

**Published:** 2021-08-27

**Authors:** Liang-Ming Jia, Fang-Wu Tung

**Affiliations:** Department of Design, National Taiwan University of Science and Technology, No.43, Keelung Rd., Sec.4, Da’an Dist., Taipei City 106335, Taiwan; D10810801@mail.ntust.edu.tw

**Keywords:** wrist wearables, Kansei engineering, multidimensional scaling, factor analysis, triangular fuzzy theory, general linear model

## Abstract

This study aimed to investigate consumers’ visual image evaluation of wrist wearables based on Kansei engineering. A total of 8 representative samples were screened from 99 samples using the multidimensional scaling (MDS) method. Five groups of adjectives were identified to allow participants to express their visual impressions of wrist wearable devices through a questionnaire survey and factor analysis. The evaluation of eight samples using the five groups of adjectives was analyzed utilizing the triangle fuzzy theory. The results showed a relatively different evaluation of the eight samples in the groups of “fashionable and individual” and “rational and decent”, but little distinction in the groups of “practical and durable”, “modern and smart” and “convenient and multiple”. Furthermore, wrist wearables with a shape close to a traditional watch dial (round), with a bezel and mechanical buttons (moderate complexity) and asymmetric forms received a higher evaluation. The acceptance of square- and elliptical-shaped wrist wearables was relatively low. Among the square- and rectangular-shaped wrist wearables, the greater the curvature of the chamfer, the higher the acceptance. Apparent contrast between the color of the screen and the casing had good acceptance. The influence of display size on consumer evaluations was relatively small. Similar results were obtained in the evaluation of preferences and willingness to purchase. The results of this study objectively and effectively reflect consumers’ evaluation and potential demand for the visual images of wrist wearables and provide a reference for designers and industry professionals.

## 1. Introduction

With the COVID-19 pandemic, people are gradually becoming more focused on their health, including maintaining physical health, strengthening their immunity and preventing disease. Moreover, with the rapid spread of internet technology and lifestyle changes, the relatively low cost of personal mobile devices is attracting attention and a new developing trend has emerged—wearable devices [1,2,3]. Wearable devices are widely applied in various fields, providing great convenience for people’s lives [4]. According to the data announced by the IDC (International Data Corporation), the shipment of wearable devices exceeded 100 million units for the first time in the first quarter of 2021, reaching 104.6 million units, an increase of 34.4% from 77.8 million units in the same period last year [5]. In addition, compared to total global wearables spending (USD 69 billion) in 2020, Gartner forecasts that it will grow by 18.1% to USD 81.5 billion in 2021 and maintain a year-on-year increase in the next few years, reaching USD 109 billion in 2024 [6,7]. Wearable devices, similar to smartphones, achieve multiple functional uses for consumers, are equipped with various operating systems to assist people in continuously receiving physiological data, such as heart rate, sleep quality and body temperature, while also providing reference to other parameters of the body, accessing more health information and effective health management for themselves and improving their quality of life [3,8,9,10].

In recent years, a growing number of brands and types of wearable devices have appeared on the market and the functions are gradually diversifying, providing consumers with a wealth of choices [2]. Among the various wearable devices, wrist wearables stand out as a significant contributor to the wearable devices market [11]. According to the shipment data published by Counterpoint Research in 2021, wrist wearables accounted for 41% of the whole wearables market (wrist, ear, body, eye and skin) in the third quarter of 2020 and will probably keep increasing their proportion in the market [12]. In addition, with the arrival of the post-epidemic era, the gradual normalization of the economy and the growth of people’s health awareness will also help the development of the wrist wearables market. As shown by the latest statistics from Canalys, the number of wrist wearables sold in 2020 reached 185 million units, an increase of 10%, showing a considerable upswing, and, in 2021, it is expected that the same trend of development will be maintained, possibly surpassing 200 million units for the first time [13].

Besides the functional elements, the aesthetic experience generated by the visual images of wrist wearables also profoundly impact consumers’ psychology [14,15]. Wrist wearables are fashionable and are regarded as an accessory indicating personal taste and identity [16]. The design aesthetics of wrist wearables is an essential factor that attracts the attention of consumers and increases their willingness to adopt them [17,18]. When consumers purchase products, they inject their emotions into the products that capture their attention; thus, emotions are one of the significant factors affecting consumers’ purchasing behavior [19]. The appearance of the product could be a competitive advantage [20], as the appearance of the product form has a positive impact on the sustainable perceived value of wrist wearables [21]. Scholars [22,23,24] noted that the appearance of wrist wearables, including color, shape and display size, provides a certain sense of design aesthetics. Several scholars have attempted to determine the visual image characteristics of the product and the reasons that affect consumers’ emotions through the conceptual model and the semantic differential (SD) method [25]. Several scholars have obtained the correlation matrix between consumers and product samples through the Kansei engineering method, then extracted the design elements of product samples analytically to provide a reference for product optimization [26,27]. As a consumer-driven method for the development of products, Kansei engineering allows consumers to translate their visual and psychological impressions of a product into design elements [28]. The addition of MDS provides a more objective and effective method for researchers to perform representative sample screening. The fuzzy theory can then be used to quantify the data from participants’ evaluations of representative samples into objective, helpful information. This helps industry professionals to design products that correspond to the sentimental requirements of the consumers, which, in turn, affects their willingness to purchase [29].

The future development and application of wrist wearables is showing a trend of continuous expansion. Most of the current research on wrist wearables focuses on functional elements, manufacturing processes, communication technologies, system development and data analysis [30,31,32], while there have been few studies on the connection between consumer emotions and the appearance of wrist wearables [27]. More studies can contribute to a better understanding of consumers’ perceptions of wrist wearables. Therefore, this study applied Kansei engineering, multidimensional scaling (MDS) and triangular fuzzy theory to objectively investigate consumers’ visual evaluation of the appearance of wrist wearables. The aims of this research are therefore as follows: (1) to find representative samples of wrist wearables and determine the appropriate visual image adjectives for wrist wearables and group them accordingly, (2) to name the groups and use them to investigate consumers’ visual image evaluation of these representative wrist wearables, (3) to investigate consumers’ preferences and willingness to purchase the selected representative wrist wearables and to verify the results with consumers’ visual image evaluation and (4) to provide a reference model and process for design researchers in this field. 

The structure of this paper is as follows: Section 1 explains the research background and objectives and introduces relevant literature on the research issues. Section 2 elaborates the research methodology and theories involved in this study. Section 3 presents the framework of the research process and the steps for practical implementation. Section 4 presents and discusses the results of the study. Section 5 provides the conclusion, suggestions and the implications of the study, while Section 6 notes the research limitations and future study directions.

## 2. Theoretical Background

**Figure 1:**
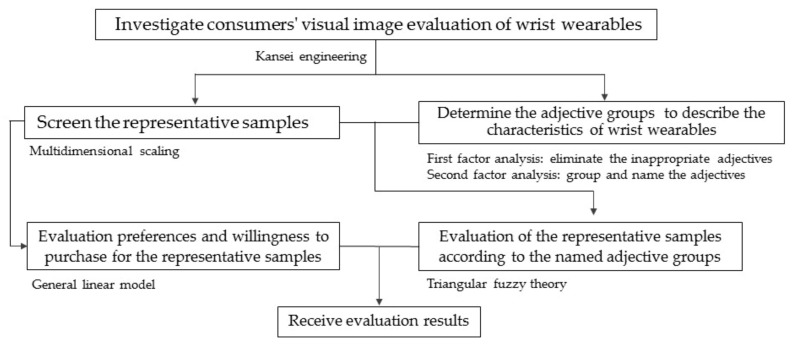
The research framework of this study.

The research methods and theories covered in this paper include Kansei engineering, multidimensional scaling (MDS), factor analysis, fuzzy theory and general linear model. The research framework is shown in Figure 1.

### 2.1. Kansei Engineering

Kansei engineering is one of the major research areas in ergonomics [33]. Kansei engineering was developed as a consumer-oriented technique to understand customers’ emotional responses and better translate them into product design elements [34,35]. In Kansei engineering, consumers typically employ adjectives (kansei words) to describe their perceptions of a product [36]. In general, Kansei engineering studies are conducted according to the following four steps [33]: (1) Selection of a product domain and collection of Kansei words and product images of study subjects from various sources—typically, more than 50 sense words and samples are collected and a reasonable reduction is made [37]. Inviting experts to conduct screening is one of the effective methods. In this step, MDS is planned to be used for the screening of representative samples. (2) Semantic space spanning—Kansei words are usually filtered using factor analysis, cluster analysis, or other methods to make the semantic space more rigorous. As the adjectives collected are independent, factor analysis is more appropriate in this study [33]. (3) Properties space spanning—the shape characteristics are usually defined graphically because it is straightforward for consumers to comprehend complex shapes and patterns [38]. The samples and Kansei words are typically combined with a 7-point Likert scale to divide the products and build the questionnaire [33]. (4) Relationship model building—this step requires associating the properties space with the semantic space. Commonly used approaches are multiple regression analysis, artificial neural networks and so on. In this step, the results are planned to be analyzed by triangular fuzzy theory.

### 2.2. Multidimensional Scaling (MDS)

MDS is a computing technology that is used to visualize data information [39,40]. In contrast to attribute-based approaches such as factor analysis, MDS can develop a perceptual map [41]. It is also a valuable way to explore the relationships in data sets [42]. By plotting the items in a low dimension space, the researchers can quickly analyze probable relationships that are difficult to solve in higher dimensions [39]. The algorithm could improve the recognition efficiency [43]. The primary function is to detect potential dimensions that enable the researchers to visualize the similarities among the studied items [44]. In other words, MDS can be applied to analyze data with the concept of distance (dissimilarity data) to show the degree of dissimilarity between things and transform them into a spatial structure while preserving the relative distance between them and is a method that can be used to reveal consumers’ preferences for a certain type of product [45,46,47]. One of the most significant advantages of MDS is that it allows restricting the perceived dimensions of perception to a range of stimuli and does not require a large number of samples to obtain reliable results [48]. When selecting the representative samples for research by market share or sales ranking [26,27,49], some samples with a high similarity may occur, which leads to a decrease in the representativeness of the samples. The results obtained by this method are more objective and rigorous. The stress index is often accepted as an indicator of quality and appropriateness in the selection of samples [46]. In this study, Kruskal and Wish’s (1978) criteria were used and the stress index ranged from a value of 0 to 1, where the lower the stress index, the more suitable it was. Specifically, the stress index is 0.200 for poor, 0.100 for fair, 0.050 for good, 0.025 for excellent and 0.000 for perfect [46,50]. 

### 2.3. Factor Analysis

Factor analysis is a statistical method that simplifies complex data by replacing the original data structure with fewer dimensions to reflect the inner nature, state and characteristics of things [26,51,52,53]. The characteristics of each dimension are defined through the measurement of the content and commonality of the data contained in each dimension [52,54]. Replacing the original variables with these factors can effectively decrease the overall complexity of the extensive data [55]. The factor loadings are usually the correlation coefficients of the variables with the factors [56]. The factor loadings range from −1 to +1. The larger the absolute value, the stronger the relationship between the corresponding factor and the variable [55]. The factor loadings indicate the variance explained by the variable for that particular factor, while the eigenvalues supply the variance explained by that specific factor in the total variance [57]. According to Kaiser’s study, a KMO value closer to 1 indicates a higher degree of variance correlation, making it more appropriate for factor analysis [58]. 

### 2.4. Fuzzy Theory

Fuzzy theory, first proposed by Zadeh in 1965, is a discipline that deals with and investigates fuzzy phenomena, with the aim of solving the problem of ambiguity and vagueness in the real world by mathematically transforming indeterminate conceptual language into a mathematical form [59]. The process of cognition is inherently fuzzy. In order to deal with these fuzzy things, fuzzy sets and fuzzy membership functions can be used to quantify the uncertain data into usable information through a systematic fuzzy computation process [60,61]. The fuzzy theory extends traditional mathematics from a relative binary logic to a continuous multi-value logic with gray areas, where the characteristic value of an element belonging to a set is no longer 0 or 1, but a numerical value which expresses the degree of the set (typically a value ranging from 0 to 1) [26,62,63,64]. Fuzzy theory has been widely applied in various research fields and is frequently employed in the design research field [65,66].

Triangular fuzzy numbers, trapezoid fuzzy numbers and bell-shaped fuzzy numbers are the common forms of membership functions and the fuzzy number to be adopted for the operation depends on the research demands [67]. In this study, the most commonly used triangular fuzzy number was used to express product attributes. The distribution of probabilities is a triangle, which can improve the accuracy, completeness and practicality of interval estimation [60,68,69]. Assume a triangular fuzzy number in $\tilde{t}$ functions as $\mu_{i}\left(x\right)$ which is $\tilde{t}$ = $\left(t_{1},t_{2},t_{3}\right),{\;\mathrm{where}\;t}_{1},{\;t}_{2},{\;\mathrm{and}\;t}_{3}$ are real numbers and $t_{1}\leq t_{2}\leq t_{3}$ [70].

**Figure 2:**
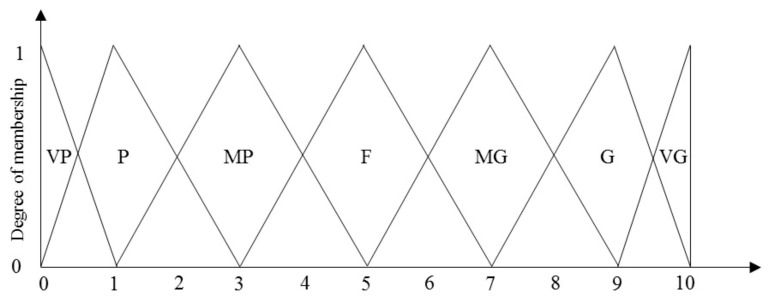
The membership function of the triangular fuzzy number.

As shown in Table 1, seven levels (1 = very poor, 7 = very good) of fuzzy meaning were adopted as a ranking method in this study [71]. The results of fuzzy ranking were obtained through a 7-level membership function, which quantified the fuzzy meaning to obtain the triangular fuzzy number [26,72]. The triangular fuzzy number of the membership function is shown in Figure 2.

Following the acquisition of the triangular fuzzy numbers, this study proposed to apply the method presented by Chen in 1985, which aimed to defuzzify the triangular fuzzy numbers in the affiliation function using the maximization set and the minimization set and the total utility or order value of the sample is determined by the weights of these two triangular fuzzy numbers [73,74]. The specific calculation is as follows: assuming that there are n triangular fuzzy numbers in a membership function, it is described as ${\tilde{t}}_{i}=\left(t_{1},t_{2},t_{3}\right)$, i = 1, 2, …, n; the maximum $\mu_{M}\left(x\right)$ and minimum $\mu_{G}\left(x\right)$ in this study are respectively M and G [63]. The absolute utility value of $U_{T}$ of the triangular fuzzy number $\left({\tilde{t}}_{i}\right)$ is as Equation (1) shows:

(1) $$U_{T}\left({\tilde{t}}_{i}\right)=\left[\frac{\left(t_{i3}-X_{\min}\right)}{\left(\left(X_{\max}-X_{\min}\right)+\left(t_{i3}-t_{i2}\right)\right)}+1-\frac{\left(X_{\max}-t_{i1}\right)}{\left(\left(X_{\max}-X_{\min}\right)+\left(t_{i2}-t_{i1}\right)\right)}\right]/2,\;i=1,\;2,\ldots ,\;n$$

### 2.5. General Linear Model

ANOVA can show where the differences between experimental groups occurr [75]. This study adopted the general linear model for one-way ANOVA to evaluate the differences in the consumers’ preferences and the willingness to purchase. After achieving statistical significance, LSD post-hoc intergroup tests were performed [76].

## 3. Implementation Procedures

**Figure 3:**
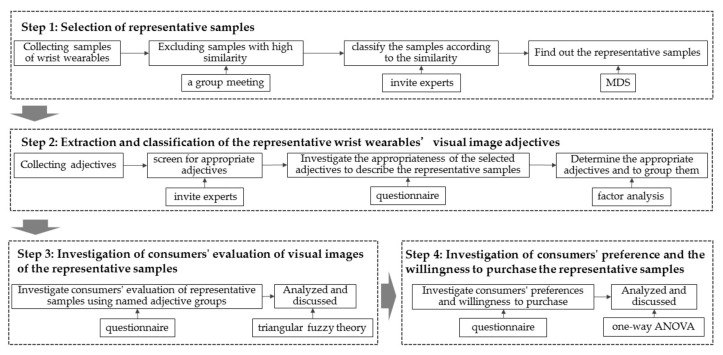
The implementation procedures of this study.

On the basis of the theory described in Section 2, the framework of this study is shown in Figure 3 and the implementation steps are as follows: first step, selection of the representative samples (by MDS); second step, extraction and classification of the visual image adjectives of the representative samples (by factor analysis); third step, investigation of consumers’ evaluation of visual images for the representative samples (by fuzzy theory); fourth step, investigation of consumers’ preference and willingness to purchase the representative samples (by general linear model). 

### 3.1. Step 1: Selection of the Representative Samples

**Figure 4:**
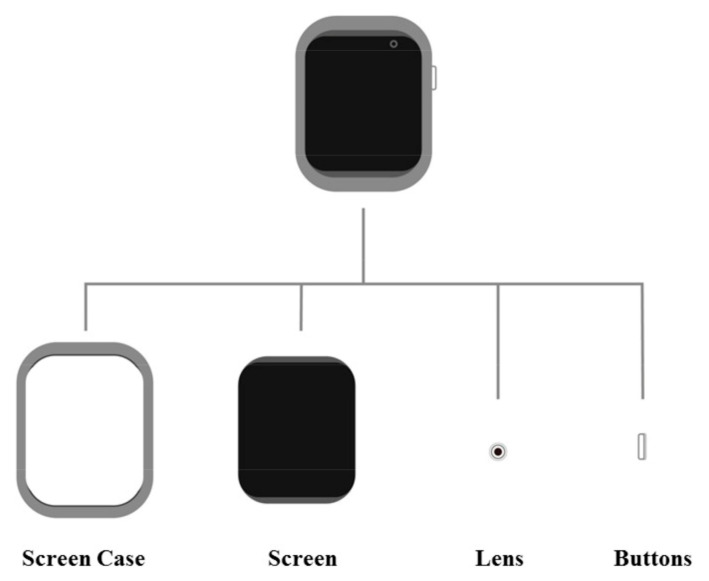
The morphological characteristics of the wrist wearables.

**Figure 5:**
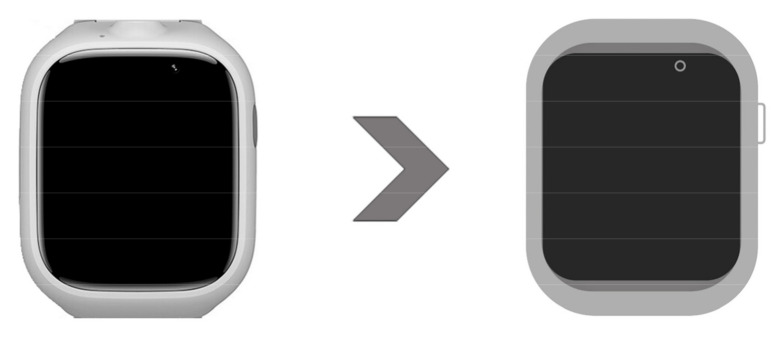
2D image transformed from the front view of the samples.

The samples selected for this research were obtained from various wrist wearable device sites, shopping sites, search engines and magazines. A total of 115 front images of wrist wearables with clear background and no miscellaneous information interference were collected as samples. Due to the presence of samples with high similarity among the collected samples, the research team conducted a five-person focus group meeting to reduce the number of samples to provide to experts for classification; ultimately, 99 samples were left. These samples were then coded. A total of 12 specialists conducted the experiment (including 5 design professionals with more than 5 years of professional experience, 2 lecturers from university design departments and 5 Ph.D. design students); they were asked to categorize (7–15 categories) the similarities of the appearance characteristics (see Figure 4) of the provided samples. The research team created a similarity matrix based on the results of the expert classification. After transforming it into a dissimilarity matrix, MDS analysis was performed to obtain the values of the dimensions of the samples, stress and RSQ. The values of each sample in the obtained best dimensions were taken as variables for cluster analysis and the samples were categorized by cluster analysis. The distance of each sample to the centroid of its category was obtained and the closest to the centroid was the representative sample of each category. The research team worked on the semantics and shape of product appearance in this study. The Adobe Illustrator CC 2017 for win software was used to transform the front view of the represented samples into two-dimensional figures (to avoid the influence of the brand, material, etc.) in the next step, as shown in Figure 5.

### 3.2. Step 2: Extraction and Classification of the Visual Image Adjectives of the Representative Samples

The research team searched for adjectives related to visual images of wrist wearables on various wrist wearable websites, shopping sites, search engines and magazines in Taiwan. After a group meeting of the research team, 120 adjectives were selected for the next step of the expert questionnaire survey. A total of 19 experts (including 2 university design teachers, 1 high school Chinese teacher, 5 Ph.D. students in Chinese literature, 6 Ph.D. students in design and 5 MA students in design) were invited to participate in the experiment. They were required to select the adjectives they thought adequately represented the appearance of these wrist wearables. A total of 50 adjectives were selected and ranked according to their selection frequency and the 40 most frequently selected were used in the next step of the questionnaire. A 5-point Likert scale questionnaire (1 = strongly disagree, 5 = strongly agree) was used to investigate consumers’ overall appropriateness of the 40 adjectives selected to describe the appearance of the representative samples (e.g., “Do you think modern is an appropriate adjective to describe the appearance of these samples?”). The research team conducted the first factor analysis on the questionnaire results to extract and classify the visual image adjectives for the representative samples. Liu et al. categorized the factor loading into strong, medium and weak levels, which corresponded to >0.75, 0.75–0.50 and 0.50–0.30 [77]. The adjectives with factor loadings below 0.5 were eliminated to obtain adjectives with more relevance. Then, the second factor analysis was conducted and the component matrices grouped the adjectives. Finally, the groups were named according to the characteristics associated with the adjectives in the group.

### 3.3. Step 3: Investigation of Consumers’ Evaluation of Visual Images for the Representative Samples

A questionnaire conducted to investigate consumers’ evaluation of the representative samples. The questionnaire was designed according to the principle of fuzzy meaning, with a 7-point Likert scale (1 = strongly disagree, 7 = strongly agree) corresponding to seven levels of fuzzy meaning, where each representative sample was matched with each group of named adjectives (results from step 2). For example: “How do you evaluate the fashionable and individual features of the appearance of S59 (S means sample)?”, “How do you evaluate the rational and decent features of the appearance of S59?” and so on.

### 3.4. Step 4: Investigation of Consumers’ Preference and the Willingness to Purchase the Representative Samples

This step was also conducted through a questionnaire to investigate consumers’ preferences and willingness to purchase the representative samples to verify step 3 in another way. The research team put the related questions in the questionnaire of step 3 using a 7-point Likert scale (1 = strongly disagree, 7 = strongly agree). For example: “To what extent do you prefer S59?”, “What is the extent of your willingness to purchase S59?”, etc.

## 4. Results and Discussion

### 4.1. Screening Outcomes of the Representative Wrist Wearable Samples

As shown in Table 2, the best results were obtained with six dimensions by MDS with the stress value of 0.05685 and the RSQ value of 0.94614. The stress value was close to 0.050, which indicates that the quality and appropriateness of the samples are good.

**Figure 6:**
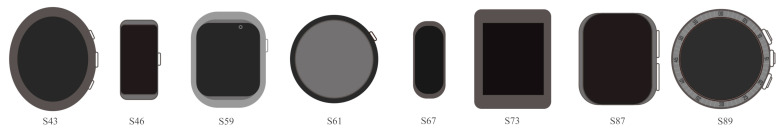
The eight representative samples.

The research team used the six dimensions (obtained by MDS) of the 99 samples as variables for clustering analysis. By the cluster analysis, the 99 samples were classified into eight groups and obtained the distance from each sample to the center of its category. The sample nearest to the center of the category was the most representative in that category, as shown in Table 3. The eight representative samples are shown in Figure 6. The representative samples screened by MDS were partially similar to the results of Zhao et al. [27] and Wan and Hsu’s study [49], which included a few samples with good sales volume and some less common but equally representative samples.

### 4.2. The Results for Visual Image Adjective Extraction and Classification

The 19 experts were invited to select the appropriate adjectives (according to the appearance) to describe these eight representative wrist wearables. A total of 50 adjectives was selected. The research team then compiled the 40 most frequently selected adjectives for further analysis, as shown in Table 4.

Then, the questionnaire was conducted by combining the 40 most recognized adjectives with eight representative samples (e.g., “Do you think modern is an appropriate adjective to describe the appearance of these samples?”). In total, 144 questionnaires were collected by means of convenience sampling, of which 122 were valid (67 male and 55 female). The data were subjected to the first factor analysis, then the component analysis was performed and the factors with the factor loading higher than 0.5 (32 in total), as shown in Table 5, were selected to conduct a second factor analysis.

A KMO value of 0.866 was obtained after the second factor analysis, as well as the Bartlett’s test result of 2245.702 (df = 496, *p* = 0.000), which achieved statistical significance. This result indicates that the related matrices in the original group have factors in common and are suitable for factor analysis.

Total variance shows (see Table 6) that the values of five factors were greater than 1 and the accumulative percentage of variance was 60.367% (in general, the accumulative percentage of variance greater than 60% is considered appropriate). As shown in Table 7, from the transformed matrix, the second factor analysis yielded extremely distinctive differences among the five components, which did not overlap with the others. Thus, all of the 32 adjectives and the five component factors from the factor analysis on this occasion could be utilized for the forthcoming analysis.

Through the second factor analysis, five groups consisting of the 32 adjectives were created. The research team named the groups according to the characteristics and the factor loading associated with the adjectives in the group [26,27,29]. They were used to elaborate the imaginative evaluation of the shape characteristics of these eight representative samples. They are respectively “fashionable and individual”, “rational and decent”, “practical and durable”, “modern and smart” and “convenient and multiple”, as shown in Table 8.

### 4.3. The Results of the Fuzzy Operation

The evaluation of the visual image questionnaire was designed based on the eight representative samples (as in Figure 6), combining the five groups of visual image adjectives (as in Table 8) according to the seven-level fuzzy meaning (see Table 1) corresponding to the 7-point Likert scale (e.g., “How do you evaluate the fashionable and individual features of the appearance of S59?”). A total of 130 questionnaires were collected by means of convenience sampling, of which 112 were valid. Among the participants, 46.4% (*n* = 52) were male and 54.6% (*n* = 60) were female. The highest proportion was aged 21–30 (57.1%, *n* = 64), followed by 31–40 (32.1%, *n* = 36), and 93.6% (n = 105) had a university education or above. The most predominant occupation was student (36.6%, *n* = 41), followed by professional (doctors/lawyers/athletes/journalists/teachers, etc.) with 23.2% (*n* = 26).

**Figure 7:**
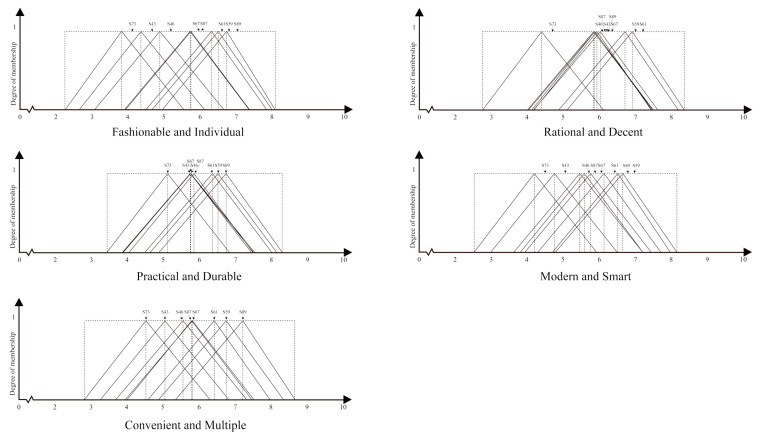
Diagrams of the triangular fuzzy number for the eight wrist wearables in the five groups of visual ratings.

The results of the questionnaire were transformed from the fuzzy meanings to the triangular fuzzy numbers by means of the triangular membership function. Then, the values were averaged by summing, as shown in Table 9. Finally, the triangular fuzzy diagrams were plotted based on the participants’ evaluation of the visual images for the eight representative samples, as shown in Figure 7.

By defuzzifying the triangular fuzzy number of each sample in Table 9 in terms of the formula for the absolute utility values, as shown in Table 10, the values of absolute utility of the visual image evaluation for the eight representative samples were obtained.

As Table 10 shows, S89 obtained the highest scores in the group of “fashionable and individual”, “practical and durable” and “convenient and multiple” S61 obtained the highest scores in the group of “rational and decent” and S59 obtained the highest scores in the group of “modern and smart”.

**Figure 8:**
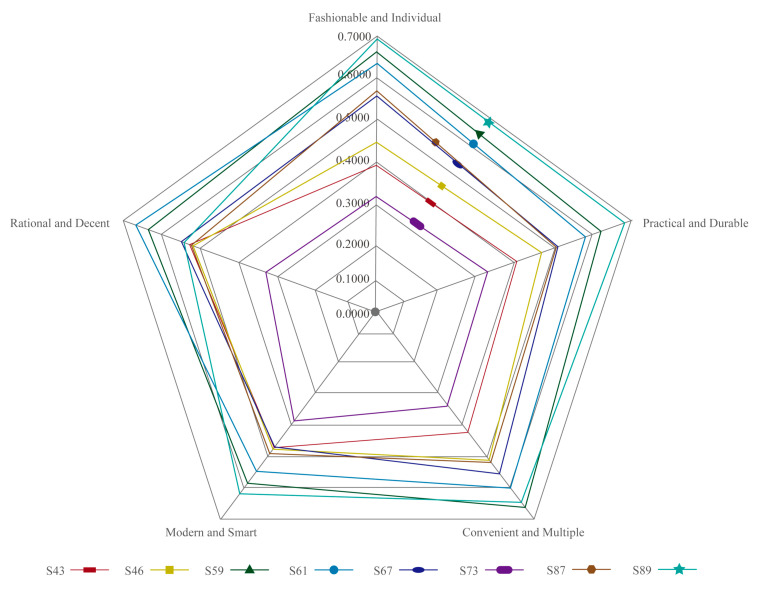
The radar plot of visual image evaluation of the eight representative wrist wearable samples.

Figure 8 shows that, following the further processing of the radar map, different wrist wearables received relatively diverse visual image evaluations in the groups of “rational and decent” and “modern and smart”. In the groups of “fashionable and individual” “practical and durable” and “convenient and multiple”, the differentiation is relatively small. The scores of visual image evaluation were combined for a comprehensive comparison and four categories were obtained; S89, S59 and S61 were placed in the outer circle of the linear radius and the visual image evaluation scores were higher, which were generally satisfactory. The linear radii of S87 and S67 were similar and were located in the central outer part, without extreme high or low points and the evaluation scores of each visual image were higher than the median value, which was relatively satisfactory on the whole. S46 and S43 were located in the middle inner part of the linear radius and the visual image evaluation score was relatively common; the overall satisfaction level was average. S73 was located in the innermost part of the linear radius; the visual image evaluation value was less satisfactory. 

A few samples with high sales volume in the market did not receive a high visual evaluation in this study. The different appearance of wrist wearables can produce distinct emotional responses [24]. S61 and S89, as two of the three wrist wearables that received the best visual evaluation, inherited the round shape of traditional watch dials in appearance [49]. This finding supports the research demonstrated by Bar and Neta that, when people are confronted with round and angular items, they always prefer the objects with rounded contours [78]. The rounded appearance of the screen of the wrist wearables can be used as an aesthetic implication to inspire consumers’ aesthetic sensibilities [21]. In addition, S59, S61 and S89 all have mechanical buttons and bezels as components, making them slightly more complex than the other samples. This finding is similar to the research by Berlyne [79] and Creusen et al. [80], in that consumers prefer items with moderate complexity. S59, S61 and S89 are not entirely symmetrical in appearance, compared to S67 and S73. This finding supports the study by Luffarelli et al. [81] and Leder et al. [82] that asymmetrical visual stimuli attract more attention than symmetrical ones and is contrary to the findings of Tinio et al. [83]. S59 was the only wrist wearable with a lens in the eight representative samples, which was probably one of the essential reasons for bringing consumers a modern and smart visual experience. The evaluations of S73, S87 and S59 increased in this order and, interestingly, so did the curvature of their chamfers. It can be guessed that, in square shapes, the greater the curvature of the chamfer of the wrist wearable, the greater the acceptance. S67 was evaluated as being better than S46 in most groups. Similarly, S67 had a higher chamfer curvature than S46, so it can be guessed that, in rectangular wrist wearables, the greater the chamfer curvature, the better the evaluation. S43 and S47 were the representative samples with the lowest ratings, indicating that participants’ acceptance of wrist wearables with a shape close to square or ellipse was relatively low. The three samples S59, S61 and S89 with better evaluations had more obvious differences in the color of the screen and the casing and the contrast among the colors may have been one of the factors affecting the consumers’ evaluation. In addition, we could also find that participants’ evaluations of wrist wearables with similar display sizes were different (e.g., S46 and S67; S59 and S87) and evaluations of wrist wearables with different display sizes were similar (e.g., S59 and S87); thus, we inferred that the influence of display size on consumer evaluations was relatively small.

### 4.4. The Results and Discussion of the Preferences

From the outcomes presented in Table 11, there was a significant difference in preferences among the participants for different wrist wearables ($F_{\left(\text{7,888}\right)}$ = 19.650, *p* = 0.000). The specific differences between the samples are shown from the results of the post-hoc tests (i.e., LSD). Specifically, the participants’ preferences for S46, S67 and S87 were significantly higher than those for S43 and S73, while being significantly lower than those for S59, S61 and S89. There was no significant difference in the participants’ preferences for S43 and S73. The participants’ preferences for S59, S61 and S89 did not differ significantly. The participants’ preferences for S46, S67 and S87 showed no significant difference.

According to the mean results, participants showed the highest relative preference for S59 (*M* = 4.86, *SD* = 1.17), S61 (*M* = 4.71, *SD* = 1.45) and S89 (*M* = 4.96, *SD* = 1.34). S46 (*M* = 3.99, *SD* = 1.52), S67 (*M* = 4.30, *SD* = 1.46) and S87 (*M* = 4.31, *SD* = 1.51) showed a moderate preference and were in the middle of the range. Participants’ preferences for S43 (*M* = 3.43, *SD* = 1.59) and S73 (*M* = 3.36, *SD* = 1.61) were relatively low.

In addition, in combination with the results of the fuzzy operation, as in Section 4.3, S89 received favorable evaluation of the four groups except for “rational and decent”, while there was no significant difference in preference for S59 and S61. S43 received relatively negative ratings except for “rational and decent” and “practical and durable”, while there was no significant difference between S73 and S43 in preference. Therefore, “fashionable and individual”, “modern and smart” and “convenient and multiple” may be the factors that affect consumers’ preference for wrist wearables. The above results are similar to Wang and Hsu’s study [49]; the round shape of wrist wearables is more recognizable and acceptable to consumers than the square shape, eliciting a more positive emotional response. It is also more probable to achieve support for symbolic and identity-related purposes [24]. In addition, it also expands Wu’s study that young and middle-aged groups prefer asymmetrical forms [84].

### 4.5. The Results and Discussion of the Willingness to Purchase

From the outcomes as presented in Table 12, there is a significant difference in willingness to purchase among the different wrist wearables ($F_{\left(\text{7,888}\right)}$ = 19.213, *p* = 0.000). The specific differences between the samples are shown from the results of the post-hoc tests (i.e., LSD). Specifically, the participants’ willingness to purchase S46 and S67 was significantly higher than that of S43 and S73, while significantly lower than that of S59, S61 and S89. The participants’ willingness to purchase S43 and S73 was significantly lower than that of S59, S61 and S87. The participants’ willingness to purchase S43 and S73 was significantly lower than that of S67 and S87. The participants’ willingness to purchase S87 was significantly higher than that of S46, while significantly lower than that of S89.

Based on the results of the averages, participants had relatively the highest willingness to purchase S59 (*M* = 4.72, *SD* = 1.42), S61 (*M* = 4.71, *SD* = 1.40) and S89 (*M* = 4.81, *SD* = 1.40), while there was no significant difference in the evaluation of these three samples. There was relatively high willingness to purchase S87 (*M* = 4.32, *SD* = 1.56), which was significantly less than S89 and not significantly different from S59 and S61. Participants showed moderate purchase intentions for S67 (*M* = 4.11, *SD* = 1.58) and S46 (*M* = 3.72, *SD* = 1.60) and there was no significant difference between the two samples. Participants were relatively less willing to purchase S43 (*M* = 3.30, *SD* = 1.68) and S73 (*M* = 3.22, *SD* = 1.70) and there was no significant difference between the two samples.

In addition, the above results also basically echo the results of the fuzzy operation and the participants’ preference evaluation of these eight wrist wearables. The results above are consistent with Hsiao and Chen’s [85] study that consumers’ evaluation of the appearance of wrist wearables influences their willingness to purchase. In addition, a few of the better-selling wrist wearables in the samples did not receive a high level of purchase intent, indicating that appearance may not be a decisive factor influencing consumers’ purchase behavior.

## 5. Conclusions and Suggestions

### 5.1. Conclusions

Based on Kansei engineering, this study integrated MDS and fuzzy theory methods to investigate consumers’ visual evaluation of the appearance of wrist wearables.

In terms of theoretical implications, this study contributes to the research on the appearance of wrist wearables. It provides a model for the researcher to refer to when conducting this kind of research. Kansei engineering is a method of exploring consumers’ emotional response to a particular product category [34,35], but the addition of MDS provides a more objective and effective method for researchers to perform representative sample screening. The fuzzy theory is then used to quantify the data from participants’ evaluations of representative samples into objective, helpful information [60,61]. This paper also compared the results with previous studies [21,24,49,78,79,80,81,82,83,84,85]. In this study, wrist wearables with a shape close to a traditional watch dial (round), with a bezel and mechanical buttons (moderate complexity) and asymmetric forms may receive a higher evaluation, preference and willingness to purchase. Among the eight representative sample shapes, square and ellipse are relatively less well accepted, followed by rectangular, with round being the best. For square and rectangular appearing wrist wearables, the greater the chamfer, the greater the acceptance. The contrast between the color of the screen and the casing may be one of the factors that affect the consumer’s evaluation, where the more apparent the contrast, the higher the evaluation. In addition, the influence of display size on consumer evaluations was relatively small. This study provides empirical evidence of the relationship between the appearance of wrist wearables and consumers’ visual evaluation.

In terms of practical implications, the participants’ impressions, psychological feelings, preferences and purchase intentions regarding the appearance of wrist wearables were understood. A total of eight representative samples and five groups of adjectives were compiled. The results of the survey showed that there was a significant difference between “rational and decent” and “modern and smart”. The aesthetic study of the appearance of wrist wearables could be helpful for enterprises or companies to improve or create the appearance attributes of new products to cater to the present market demand of differentiation and precision and propose timely and differentiated marketing strategies. In addition, the findings of this study reveal the impact of wrist wearables’ appearance attributes on consumers’ perceptions, preferences and willingness to purchase, which could help designers establish the perceptual connection between products and consumers, thus attracting consumers more effectively.

### 5.2. Suggestions

In terms of methodology, Kansei engineering has been used extensively in the area of design research. MDS can be used to screen representative samples in such studies, which helps to improve the objectivity and representativeness of the selected samples. The fuzzy theory could also be used to interpret consumers’ evaluation of a specific type of product.

In terms of study results and participants, the representative samples in this study have relatively limited differentiation among “fashionable and individual”, “practical and durable” and “convenient and multiple”. It is possible to strengthen the distinction among these three groups of factors in designing future wrist wearables and making targeted upgrades to the products. Moreover, the participants in this study were mainly aged 21–40 years (89.2%), had university and above education (93.6%) and were students and professionals (59.8%). S89, S61 and S59 received relatively high evaluations in general. Therefore, these three relatively highest evaluated samples can be referred to when conducting future design or marketing processes for the participant groups in this study. Meanwhile, square and elliptical shapes should be minimized in the design of the wrist wearable. In addition, in the design of the square and rectangular wrist wearables’ appearance, the curvature of the chamfer could be expanded appropriately. It could also be designed to enhance the contrast between the screen and the case.

## 6. Research Limitations and Future Directions

The results of this study have a degree of generalizability limitation. First of all, to control for variables, the main object of this study is the appearance of wrist wearables and the factors that influence consumers’ evaluations, preferences and purchase intentions include several others. Therefore, in the future, different materials, brands, or operating systems could be considered as research variables to explore their differences. 

Secondly, the participants in this study were mainly from Chinese regions. Owing to the differences in region, culture and life, the study results may be limited to that region and cannot be extended to others. In the future, different cultural regions can be used as considerations to explore various aesthetic preferences and emotional demands.

In addition, the subjects in this study were primarily middle-aged and young adults, which is not quite sufficient for the generalizability of the study results. Future studies could be conducted for different genders, age groups and occupations to enhance the generalizability of the study results.

Finally, the samples in this study are in the form of images, which may not bring the most intuitive feeling to the subjects. In the future, the representative samples of wrist wearables can be transformed into an entity for the survey so that the participants can simulate the actual situation as much as possible for evaluation and enhance the quality of the study.

This study is the beginning of the research on the perception of wrist wearable appearance to consumers. Following the growing popularity of health concepts and the increasing popularity of wrist wearables in people’s lives, there is a growing need for research on this topic.

## Figures and Tables

**Table 1:** Linguistic variables for the ratings.

Linguistic Scales	Corresponding Triangular Fuzzy Number
Very poor (VP)	(0,0,1)
Poor (P)	(0,1,3)
Medium poor (MP)	(1,3,5)
Fair (F)	(3,5,7)
Medium good (MG)	(5,7,9)
Good (G)	(7,9,10)
Very good (VG)	(9,10,10)

**Table 2:** The results of the stress and RSQ values in different dimensions of MDS analysis.

**Dimensions**	2	3	4	5	6
**Stress**	0.19598	0.12567	0.08583	0.06803	0.05685
**RSQ**	0.78577	0.87374	0.92253	0.93835	0.94614

**Table 3:** The results of cluster analysis.

**Category**	1	2	3	4	5	6	7	8
**Sample**	S43	S46	S59	S61	S67	S73	S87	S89
**Distance**	0.85811	0.89645	0.41045	0.54486	0.97418	1.30389	0.56787	0.67504

**Table 4:** The 40 most recognized adjectives.

Modern	Smart	Scientific	Concise	Fashionable	Stylish	Practical	Exquisite
Accessible	Classic	Durable	Quality	Multifunctional	Superior	Fancy	Fascinating
Minimalist	Convenient	Geometrical	Decent	Magnificent	Innovative	Futuristic	Steady
Compact	Rational	Tidy	Advanced	Precise	Mechanical	Ordered	Delicate
Individual	Simple	Sleek	Trendy	Refreshing	Excellent	Unique	Mature

**Table 5:** The 32 adjectives with factor loadings higher than 0.5.

Adjectives	Extraction	Adjectives	Extraction	Adjectives	Extraction
Modern	0.601	Superior	0.683	Ordered	0.569
Smart	0.747	Fancy	0.566	Delicate	0.546
Scientific	0.659	Fascinating	0.578	Individual	0.577
Fashionable	0.542	Convenient	0.570	Simple	0.518
Practical	0.559	Decent	0.564	Sleek	0.575
Exquisite	0.633	Magnificent	0.594	Trendy	0.639
Accessible	0.669	Innovative	0.670	Refreshing	0.709
Classic	0.529	Futuristic	0.616	Excellent	0.634
Durable	0.626	Rational	0.508	Unique	0.624
Quality	0.657	Tidy	0.537	Mature	0.623
Multifunctional	0.566	Advanced	0.630		

**Table 6:** Total variance explained.

Factor of Component	Initial Eigenvalues			Squares Loading Extraction			Transformed Squares Loading		
	Total	Variance (%)	Accumulative (%)	Total	Variance (%)	Accumulative (%)	Total	Variance (%)	Accumulative (%)
1	11.387	35.583	35.583	11.387	35.583	35.583	5.765	18.017	18.017
2	2.654	8.292	43.876	2.654	8.292	43.876	5.301	16.566	34.583
3	2.235	6.985	50.860	2.235	6.985	50.860	3.720	11.624	46.207
4	1.663	5.198	56.058	1.663	5.198	56.058	2.746	8.581	54.788
5	1.379	4.308	60.367	1.379	4.308	60.367	1.785	5.578	60.367

**Table 7:** The component matrices following the transformation.

Adjectives	Component				
	1	2	3	4	5
Innovative	0.780	0.115	0.177	0.084	0.099
Individual	0.721	0.094	0.205	0.034	0.073
Trendy	0.690	0.307	0.075	0.155	0.197
Fascinating	0.679	0.024	0.111	0.320	−0.039
Unique	0.675	0.270	0.301	−0.057	0.024
Fashionable	0.649	0.174	0.216	0.197	0.072
Futuristic	0.649	0.121	0.210	0.243	0.277
Fancy	0.631	0.283	0.238	0.153	−0.091
Superior	0.554	0.250	0.213	0.324	−0.404
Advanced	0.533	0.356	0.067	0.290	0.361
Excellent	0.531	0.461	0.364	0.077	−0.032
Refreshing	0.274	0.782	0.049	0.049	0.134
Sleek	0.136	0.743	0.058	0.032	0.002
Ordered	0.195	0.703	0.180	−0.066	−0.020
Tidy	0.019	0.699	−0.005	0.113	0.188
Simple	0.158	0.671	0.002	0.176	0.109
Rational	0.009	0.636	0.193	0.142	0.213
Mature	0.347	0.616	0.313	−0.057	−0.146
Decent	0.121	0.589	0.199	0.231	−0.331
Delicate	0.324	0.554	0.288	0.177	−0.138
Magnificent	0.319	0.524	0.288	0.261	−0.257
Quality	0.182	0.029	0.778	0.128	−0.048
Durable	0.157	0.114	0.759	−0.057	0.091
Exquisite	0.372	0.116	0.637	0.105	−0.252
Practical	0.205	0.181	0.611	0.060	0.327
Accessible	0.202	0.206	0.609	0.134	0.444
Classic	0.300	0.302	0.587	0.053	−0.014
Smart	0.234	0.099	0.024	0.791	0.239
Scientific	0.200	0.161	0.124	0.760	0.019
Modern	0.125	0.122	0.042	0.754	−0.028
Multifunctional	0.188	0.058	0.042	0.471	0.551
Convenient	0.341	0.165	0.365	0.128	0.527

**Table 8:** Naming of each factor (groups of adjectives).

Factor	Factor (Group) Naming	Groups of Adjectives
1	Fashionable and individual	Innovative, individual, trendy, fascinating, unique, fashionable, futuristic, fancy, superior, advanced, excellent
2	Rational and decent	Refreshing, sleek, ordered, tidy, simple, rational, mature, decent, delicate, magnificent
3	Practical and durable	Quality, durable, exquisite, practical, accessible, classic
4	Modern and smart	Smart, scientific, modern
5	Convenient and multiple	Multifunctional, convenient

**Table 9:** Rankings and averages of the visual image evaluations for the 8 representative wrist wearable samples.

Fashionable and Individual	Rational and Decent	Practical and Durable	Modern and Smart	Convenient and Multiple
S89(5.23 7.03 8.39)	S61(5.39 7.21 8.67)	S89(4.88 6.73 8.29)	S59(5.08 6.94 8.44)	S89(5.35 7.21 8.65)
S59(4.96 6.80 8.30)	S59(5.16 7.01 8.49)	S59(4.63 6.51 8.13)	S89(5.00 6.81 8.27)	S59(4.88 6.75 8.33)
S61(4.78 6.61 8.14)	S67(4.46 6.32 7.92)	S61(4.49 6.36 8.00)	S61(4.59 6.42 8.00)	S61(4.59 6.42 7.96)
S87(4.22 6.04 7.68)	S43(4.32 6.17 7.79)	S46(3.87 5.76 7.52)	S67(4.23 6.06 7.72)	S67(3.96 5.81 7.51)
S67(4.19 6.02 7.66)	S46(4.29 6.13 7.78)	S43(3.86 5.74 7.49)	S87(4.11 5.88 7.50)	S87(4.01 5.79 7.45)
S46(3.36 5.18 6.96)	S87(4.41 6.17 7.76)	S87(4.04 5.84 7.48)	S46(3.93 5.76 7.50)	S46(3.69 5.54 7.31)
S43(2.94 4.66 6.42)	S89(4.47 6.22 7.74)	S67(3.88 5.75 7.43)	S43(3.29 5.05 6.79)	S43(3.27 5.05 6.81)
S73(2.54 4.11 5.85)	S73(3.05 4.69 6.41)	S73(3.43 5.11 6.82)	S73(2.83 4.49 6.22)	S73(2.82 4.52 6.29)

**Table 10:** The absolute utility values for the 8 representative wrist wearable samples.

Samples		Fashionable and Individual	Rational and Decent	Practical and Durable	Modern and Smart	Convenient and Multiple
S43	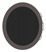	0.3950	0.5362	0.4785	0.4234	0.4094
S46	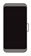	0.4618	0.5318	0.4815	0.5096	0.4725
S59	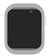	0.6689	0.6482	0.5912	0.6696	0.6270
S61	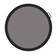	0.6444	0.6765	0.5693	0.6008	0.5837
S67	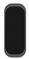	0.5684	0.5559	0.4782	0.5534	0.5061
S73	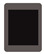	0.3239	0.3419	0.3865	0.3451	0.3412
S87	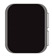	0.5713	0.5381	0.4925	0.5296	0.5043
S89	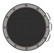	0.6992	0.5435	0.6244	0.6529	0.6864

**Table 11:** A general linear model (one-way ANOVA) for preferences.

Source	SS	DF	MS	F	*p*	η^2^	LSD
wrist wearables	294.186	7	42.027	19.650	0.000 ***	0.134	S43, S73 < S46, S67, S87 < S59, S61, S89

*** *p* < 0.001.

**Table 12:** A general linear model (one-way ANOVA) results for willingness to purchase.

Source	SS	DF	MS	F	*p*	η^2^	LSD
wrist wearables	320.929	7	45.847	19.213	0.000 ***	0.132	S43, S73 < S46, S67 < S59, S61, S89. S43, S73 < S59, S61, S87. S43, S73 < S67, S87. S46 < S87 < S89.

*** *p* < 0.001.

## Data Availability

Not applicable.
